# Risks in Antibiotic Substitution Following Medicine Shortage: A Health-Care Failure Mode and Effect Analysis of Six European Hospitals

**DOI:** 10.3389/fmed.2020.00157

**Published:** 2020-05-12

**Authors:** Nenad Miljković, Brian Godman, Eline van Overbeeke, Milena Kovačević, Karyofyllis Tsiakitzis, Athina Apatsidou, Anna Nikopoulou, Cristina Garcia Yubero, Laura Portillo Horcajada, Gunar Stemer, Darija Kuruc-Poje, Thomas De Rijdt, Tomasz Bochenek, Isabelle Huys, Branislava Miljković

**Affiliations:** ^1^Institute for Orthopaedic Surgery “Banjica”, University of Belgrade, Belgrade, Serbia; ^2^Division of Clinical Pharmacology, Karolinska Institutet, Karolinska University Hospital, Stockholm, Sweden; ^3^Strathclyde Institute of Pharmacy and Biomedical Sciences, Strathclyde University, Glasgow, United Kingdom; ^4^Department of Public Health and Management, School of Pharmacy, Sefako Makgatho Health Sciences University, Pretoria, South Africa; ^5^Clinical Pharmacology and Pharmacotherapy, KU Leuven, Leuven, Belgium; ^6^Department of Pharmacokinetics and Clinical Pharmacy, University of Belgrade, Belgrade, Serbia; ^7^General Hospital of Thessaloniki “G. Papanikolaou”, Hospital Pharmacy Department, Thessaloniki, Greece; ^8^Hospital Pharmacy Department, University Hospital “Infanta Sofia”, Madrid, Spain; ^9^Gunar Stemer, Medicines Information and Clinical Pharmacy Services, Vienna General Hospital, Vienna, Austria; ^10^General Hospital “Dr. Tomislav Bardek”, Hospital Pharmacy Department, Koprivnica, Croatia; ^11^Pharmacy Department, University Hospitals Leuven, UZ Herestraat, Leuven, Belgium; ^12^Department of Drug Management, Faculty of Health Sciences, Jagiellonian University Medical College, Krakow, Poland

**Keywords:** medicine shortage, prospective risk assessment, failure modes, Europe, hospitals

## Abstract

**Introduction:** Medicine shortages result in great risk for the continuity of patient care especially for antimicrobial treatment, potentially enhancing resistance rates and having a higher economic impact. This study aims to identify, describe, assess, and assign risk priority levels to potential failures following substitution of antimicrobial treatment due to shortages among European hospitals. Furthermore, the study investigated the impact of corrective actions on risk reduction so as to provide guidance and improve future patient care.

**Methods:** Health-care failure mode and effect analysis (HFMEA) was applied to hospitals in Austria (H-AT), Belgium (H-BE), Croatia (H-CR), Greece (H-GR), Spain (H-SP), and Serbia (H-SR). Multidisciplinary teams identified processes, failure modes, causes, and corrective actions related to antibiotic substitution following medicine shortages. Characteristics of study hospitals as well as severity, probability, and hazard scores (HSs) of failure modes/causes were analyzed using Microsoft Office Excel 2010 and IBM SPSS Statistics® via descriptive and inferential statistics.

**Results:** Through HFMEA, 74 failure modes were identified, with 53 of these scoring 8 or above on the basis of assigned severity and probability for a failure. Severity of failure modes differed before and after corrective actions in H-CR, H-GR, and H-SR (*p* < 0.005). Their probability differed in all study hospitals (*p* < 0.005) when compared before and after corrective actions aimed to be implemented. The highest number of failure-mode causes was detected in H-CR (46) and the lowest in H-SP (16). Corrective actions can address failure modes and lower HSs; therein, all teams proposed the following: structuring communication among stakeholders, introducing electronic prescribing, strengthening pharmacists' involvement, and increasing effectiveness of the ward stock assessment. These proposed actions led to HS reductions up to 83%.

**Conclusion:** There is a lack of structure in addressing risks associated with antibiotic substitution following shortages. Furthermore, lack of communication, data scarcity on availability of antibiotics, non-supportive information technology (IT) systems, and lack of internal substitution protocols hinder quick assessment of alternatives addressing patient needs. Nevertheless, the study shows that health-care professionals manage to secure optimal antimicrobial treatment for patients using available IT and human resources.

## Introduction

Medicine shortages are an everyday occurrence, causing great risk to the continuity of patient care (1, 2). Following medicine shortages, providing a suitable, clinically appropriate and safe alternative medicine to a patient is a priority to every health-care professional (3, 4). However, many risks may occur when substituting medicines including reducing the treatment effectiveness and threatening patient safety (5). These risks include, among others, incorrectly comparing the alternative medicine's administration patterns to the initial treatment, miscommunicating them to health-care professionals, and underestimating the potential for new drug–drug interactions (5).

A survey conducted by the ISMP revealed that medicine shortages pose a challenge to health-care professionals to provide safe medication treatment and avoid life-threatening medication errors, compromising the health-care process and affecting patient safety (5). The World Health Organization (WHO) Medication Without Harm initiative considers that the aforementioned errors and malpractice could be avoided through ensuring safer processes encompassing the prescription of the medicine, as well as its dispensing, preparation, and administration (6).

According to surveys from the European Association of Hospital Pharmacists (EAHP) and the American Society of Health-System Pharmacists (ASHP), antibiotics appear to be in the group of medicines most impacted by shortages (2, 7). Bearing in mind the complexity of choosing the right antimicrobial treatment in light of the looming threat of bacterial resistance (8) as has also been reported by the WHO Global report and a lack of development of new antibiotics, shortages bring even more risk to the management of antibiotic use (9–13). Significant challenges can be foreseen when considering potential consequences of the antimicrobial shortages including optimal treatment delay and an increase of unjustified use of broad-spectrum antimicrobials. This may lead to substandard therapy, resulting in inferior health outcomes and treatment efficacy and increased toxicity (10, 14).

A study conducted among health-care professionals on the perception of antimicrobial shortages in the USA found that more than 50% of professionals believe these shortages lead to the use of expensive antibiotics with lower effectiveness and safety (15). In addition, health-care professionals may not be aware of the substitute's administration and dosing patterns, possibly leading to errors in application and unexpected adverse events (16). Another problem when substituting antibiotics is the time gap between the moment of awareness of a shortage and the moment that the alternative is made available (17). Owing to the urgent nature of cases presented, a higher incidence of medication errors when a shortage occurs in emergency departments (ERs) limits the time available to account for all aspects of a substitute's application patterns. Moreover, insufficient time to relocate substitutes stored in hospital pharmacies to the ER may bear potential fatal consequences (18, 19).

In the USA, the rate of shortages of antibiotics increased from 2001 to 2013, totaling 148 antibiotic shortages being reported, including a shortage in piperacillin/tazobactam of 1,900 days (20). In Europe, the EAHP Survey on medicine shortages revealed an increase of reported antimicrobial shortages among hospital pharmacists from 57 to 77%, between 2014 and 2018 (2). Such increased antimicrobial shortages may be a factor affecting efforts undertaken by health authorities and health-care professionals to enhance the rational use of antimicrobials across health-care settings when taking into account their share in overall medicine consumption, including the use of generics (21–26).

The need to evaluate risks emerging from medicine substitution has been recognized by European, Canadian, US, and Australian health authorities (4, 27–29). This demonstrates the importance of having a risk assessment in place to prevent risks emerging from medicine substitution due to shortages. Whereas, reactive and retrospective hazard analyses such as incident reporting (IC) or root cause analysis (RCA) are focused on the event that took place in the past, proactive and prospective risk assessments such as failure mode and effect analysis (FMEA) are based on anticipating risks/hazards and proposing mitigation pathways (30). Initially developed for in the aerospace sector, the FMEA was first implemented in the 1960s. In 2002, the Veterans Affairs National Center for Patient Safety (VA NCPS) developed a modified health-care FMEA (HFMEA) version, applicable in health-care settings (30).

Several studies have recognized the importance that HFMEA has when it comes to identifying failure modes (FMs), its causes and consequences in a variety of health-care processes, such as distribution and the administration of medicines, parenteral nutrition, and chemotherapy (31–39). As recently demonstrated in a study by Castro Vida et al. (40), the HFMEA has been conducted in all processes and sub-processes in hospital pharmacies except for medicine-shortage management (40).

Consequently, we sought to address this. The aim of this study was to identify, describe, assess, and assign risk priority levels to potential failures occurring during antimicrobial treatment substitution processes in patients following shortages within health-care settings across Europe. Furthermore, the study aimed to evaluate the potential impact of proposed actions on risk reduction. This study falls under the research conducted by the wider European Cooperation in Science and Technology (COST) Action 15105-Medicines Shortages Research Network.

## Methods

### Recruitment of Hospitals

HFMEA as developed by the US Department of Veterans Affairs (VA) was applied to six European hospitals (30, 41). Hospitals were recruited via the COST Action 15105 and the EAHP networks. The HFMEA was conducted between March 2018 and April 2019 in the following hospitals: the Institute of Orthopedic Surgery “Banjica” in Belgrade, Serbia (H-SR); followed by the General Hospital “George Papanikolaou” in Thessaloniki, Greece (H-GR); the Infanta Sofia Hospital in Madrid, Spain (H-SP); the Vienna General Hospital in Vienna, Austria (H-AT); the General Hospital “Tomislav Bardek” in Koprivnica, Croatia (H-CR); and the University Hospital Leuven in Leuven, Belgium (H-BE). No ethical approval, neither written informed consent were sought for this study as the information obtained was practice-base oriented. In addition, the healthcare professionals freely participated and no patients were involved.

### Health-Care Failure Mode and Effect Analysis Conduct

The HFMEA was applied within each hospital as a prospective risk assessment tool aimed at detecting, evaluating, and ranking FMs/risks/hazards related to antibiotic substitution caused by a medicine shortage. A multidisciplinary team was created in each hospital prior to the initialization of the research. During the conduct of this study, the teams undertook an HFMEA of antibiotic substitution stemming from medicine shortages. The HFMEA team consisted of a physician, a pharmacist, a pharmacy technician, a nurse, and a person responsible for health care quality in the hospital. One member of the research team (NM; lead) was present in each hospital throughout the duration of the analysis. A dedicated member of the multidisciplinary team of each hospital (co-lead) helped in organizing study meetings. The data on the HFMEA were gathered by the lead and co-leads.

During the HFMEA, each team had to reach a consensus on the processes and sub-processes of antibiotic substitution. In addition, a hazard analysis was carried out, where FMs (possible errors) and FM causes (FMCs) were identified and scored on their severity and probability. Scores were established following a consensus procedure. According to the VA guidelines, severity was graded as catastrophic = 4, major = 3, moderate = 2, or minor = 1. Probability was scored as frequent = 4, occasional = 3, uncommon = 2, or remote = 1 (30, 41). By multiplying scores for probability and severity, a hazard score (HS) of a maximum of 16 (4 × 4) was assigned to each FM/cause. Consensus scoring prevented variability of scoring within teams as cases of differing points of view were resolved through discussion.

In the final stage, team members proposed corrective actions aiming at controlling, eliminating, or accepting detected FMCs via decision tree analysis. Corrective actions were subsequently implemented and assessed for their effectiveness through rescoring FM in all sub-processes. Depending on the HFMEA decision tree, a hazard score of 8 was considered to be a threshold: >8 were subject to further analysis and <8 were not analyzed. However, if the HS represented FMs corresponding to critical points in substituting anti-microbial treatment due to shortages (where there are no measures noted to control the hazard), the FM was further analyzed regardless of the score (30, 41).

### Analysis of Results

Information on FMs, causes, and scores was recorded in prepared paper-based templates and subsequently transferred into electronic format. The analysis followed a mixed qualitative/quantitative design, where equal attention was given to the description of processes/sub-processes and FMs/causes/effects, as well as comparison of scores. Characteristics of study hospitals and hazard analysis, as well as severity, probability, and HSs of FMs/causes were analyzed using Microsoft Office Excel 2010 and IBM SPSS Statistics® via descriptive and inferential statistics. The difference in HSs before and after corrective actions within and between hospitals was tested using Wilcoxon signed-rank test and the Kruskal–Wallis test, respectively. The statistical significance level chosen for this study was 0.05.

## Results

### Hospital Characteristics

**Figure 1:**
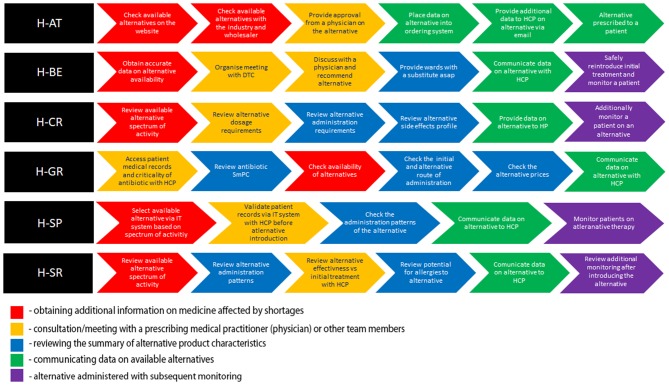
Color-coded flowchart highlighting similar patterns in sub-processes across health-care failure mode and effect analysis (HFMEA) study hospitals.

The hospitals included in this study represented a broad spectrum of large (H-AT and H-BE) and smaller hospitals (H-CR, H-GR, H-SP, and H-SR) in number of hospital beds, physicians, hospital pharmacists, and pharmacy technicians (Table 1). Although all study hospitals had Drug and Therapeutics Committees (DTCs) in place and almost all hospitals had dedicated pharmacists in charge of medicine shortages, only H-SP had a dedicated medicine shortages task force group. No hospital had internal written guidelines or mitigation pathways in place to facilitate management of shortages. All hospitals, except for H-SP, opted for six sub-processes describing antibiotic substitution in shortages (Figure 1).

**Table 1:** Characteristics of study hospitals.

**Characteristics**	**Hospitals**					
	**H-AT**	**H-BE**	**H-CR**	**H-GR**	**H-SP**	**H-SR**
Number of hospital beds	1,773	1,995	350	650	271	550
Number of physicians	1,582	1,686	149	556	368	130
Number of hospital pharmacists	33	32	3	5	8	3
Number of pharmacy technicians	64	52	5	3	14	2
Type of hospital	Tertiary care university hospital	Tertiary care university hospital	General university hospital	General university hospital	Tertiary care university hospital	Tertiary care university specialized hospital
Existing Drug and Therapeutics Committee (DTC)	Yes	Yes	Yes	Yes	Yes	Yes
Existing medicine shortages task force group	No	No	No	No	Yes	No
Existing internal guidelines on medicine shortages mitigation pathways/strategies	No	Under development	No	No	No	No
Dedicated pharmacists in charge of medicine shortages	Yes	Yes	No	Yes	Yes	Yes

### Antibiotic Replacement Sub-processes per Hospital

All hospitals, apart from H-GR, commenced the antibiotic replacement process with the assessment of the availability of alternatives (Figure 1). In H-AT and H-BE, this assessment is informed by their respective official national shortage websites. In H-SP, the availability of alternatives before feeding it within the internal hospital IT system is assessed via multiple sources including databases and manufacturers/wholesalers. H-AT confirms the availability of retrieved alternatives through oral and written communication with pharmaceutical companies and wholesalers. Health-care professionals in H-CR and H-SR focus on the antibiotic spectrum of activity when discussing possible alternative treatments. H-GR only checks availability in the third step after selecting alternatives on the basis of the patient profile, patients' clinical status, and the antibiotic spectrum.

After availability was checked, H-SR, H-SP, H-AT, H-CR, and H-BE validate patient records and the selection of the alternative with a multidisciplinary team. Health-care professionals in H-CR and H-SR compare dosage requirements, as well as reconstitution patterns, and stability issues of the alternative antibiotic treatment vs. the initial treatment as part of their decision making. In H-BE, data on all aspects of the antibiotic substitution are discussed within the hospital's DTC. In H-AT, the same manner of assessment is carried out via written/oral communication in relation to the complexity of the clinical situation in order to reach a conclusion, which can be further shared with all health-care professionals. In both H-AT and H-BE, these methods of communication are necessary prior to establishing a recommendation on an antibiotic substitute, which is then uploaded into the hospital's IT system. In H-AT and H-SP, a recommendation on the agreed substitute is subsequently communicated to all health-care professionals. In H-CR and H-SR, this transfer of data is provided only after an alternative antimicrobial adverse event profile and patient allergies are both assessed separately. Apart from health-care professionals, the information on the alternative is also provided to patients in H-CR. The need for additional patient monitoring after switching is taken into consideration in H-SR, H-SP, H-CR, and H-BE.

### Failure Modes and Failure Mode Causes per Antibiotic Replacement Sub-process

The flowcharts (Figure 1) present the processes and sub-processes that the HFMEA teams identified in a series of meetings to discuss antibiotic replacement. Throughout these meetings, the teams assigned FM (i.e., potential hazards) and FMC (i.e., potential hazard causes) to each sub-process. The teams, by consensus, assigned FM and FMC severity and probability scores and further assessed FM and FMC via decision tree analysis (30, 41).

#### Accessing Data on Alternatives

**Figure 2:**
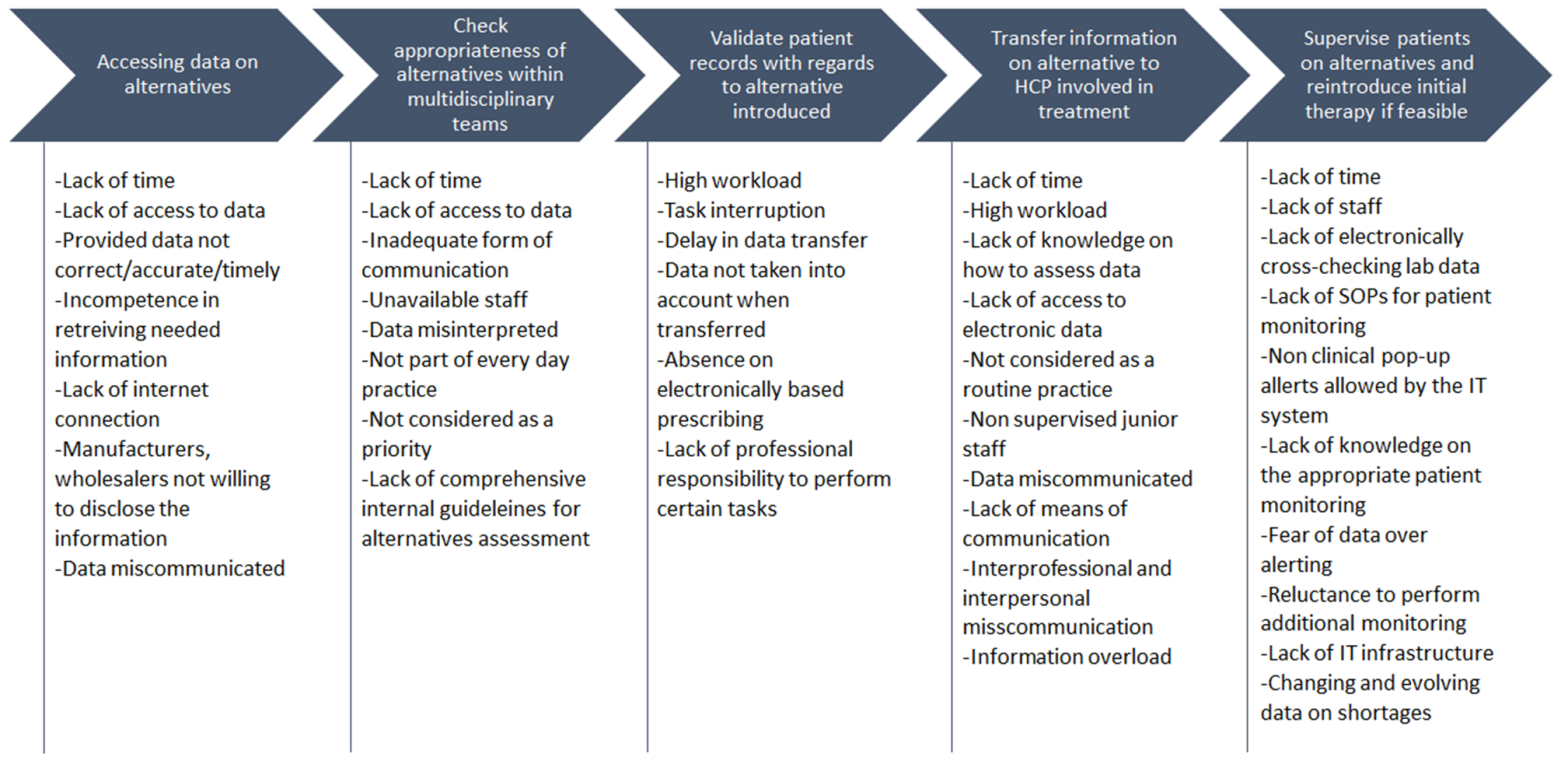
Major failure mode causes related to key sub-processes in antibiotic substitution.

The availability of alternative antibiotics is checked in H-AT and H-BE through dedicated national or health-care professional organizations websites and/or by direct contact with the wholesalers and company representatives in H-AT, H-CR, H-SP, and H-SR. FM linked to this sub-process, identified across hospitals, was a “lack of synchronization between data on alternatives availability and hospital dispensing/ordering systems.” Teams indicated that this “lack of synchronization” could be caused by “lack of time,” “failing internet connections,” “incompetence to quickly retrieve needed information,” or “getting non-accurate, non-timely or wrong/miscommunicated information” owing to manufacturers or wholesalers not being willing to disclose availability data (Figure 2).

#### Check Appropriateness of Alternatives Within Multidisciplinary Teams

When provided with data on potential available alternatives, in H-AT and H-BE, there is a structured process of sharing the data on potential antibiotic substitutes with physicians and other health-care professionals involved in mitigating medicine shortages through scheduled DTC meetings or via calls/emails, depending on the patient clinical status. However, in H-AT, approval of the substitute may not be accomplished as for “miscommunication between a hospital pharmacist and other health-care professionals” or “lack of time.” Because the risk assessment is incomplete of medicines that may be unavailable, a specific treatment regimen in H-BE for one or more groups of patients may not necessarily be taken into consideration when choosing an alternative. As mentioned by teams of H-CR and H-GR, health-care professionals involved in approving the alternative are not always available, and a substitute's dosage requirements are sometimes not well-interpreted. In H-GR, a “lack of clarity and ease to implement internal procedures/guidelines for assessment of alternatives” hinders the interpretation of them. Analysis in H-SP showed that health-care professionals' substitution assessment can fail if performed by “less experienced health-care professionals” (while senior staff are not present), “not being included into everyday practice,” or due to a “lack of time.” When it comes to agreeing on the substitute, in H-SR, comparing alternative's effectiveness with that of the initial treatment can be hampered by “lack of access to data”; alternatively, “not prioritizing the comparison of treatment effectiveness” (Figure 2).

#### Validate Patient Records With Regard to Alternative Introduced

In all HFMEA study hospitals, hospital pharmacists performed various activities within the sub-processes related to reviewing patient medical records and clinical status. This included assessing the potential for patient allergies, alternative's onset of activity, and emerging drug–drug interactions. FMs recurring here are drawn by causes such as the “lack of time,” “high workload” with “task interruption,” and “data dependency” in H-AT and H-BE. The “lack of experience and knowledge on how to efficiently assess data” and the “limited access to electronic data” are additional causes in H-SR, H-GR, and H-CR. In H-SP, previously mentioned FMs seem to be mostly caused by “senior staff routine practice not incorporating a quick change in therapy due to shortages” together with “junior staff not being aware of ongoing situations.”

#### Transfer the Information on Alternatives to Health-Care Professionals Involved in Treatment

After data on alternative antibiotic treatment are processed, along with patient record validation, this information is transferred into hospitals ordering/dispensing IT systems and disseminated to health-care professionals. Nevertheless, data transfer is sometimes delayed or not conducted properly. This is due to causes such as “high workload” connected with “task interruption” and “miscommunication or information overload” as in H-AT. Sometimes, a frequent change in alternatives availability makes it difficult to sustain a “proper data flow” as mentioned in H-BE, which is also followed by absence of obligatory ward stock replenishments on a daily basis. Moreover, a nurse in H-BE might not understand the way data are presented or does not pay attention to a message transferred. In H-CR and H-SR, apart from a “lack of time,” a “lack of means of communication” may delay antibiotic alternative data transfer. For H-GR, FMs are related to transferring information occur within the last phase of antibiotic substitution and are caused by “inter-professional and interpersonal misunderstanding” and “absence of quality systems that define communication pathways.” In H-SP, another FM was that health-care professionals that are not directly involved in prescribing antibiotics are misinformed. This can be attributed to the fact that some physicians “do not prescribe via electronic prescribing systems.” They are therefore “not aware of current shortages and proposed or expect others to deal with the issues as they feel it is not their responsibility.” Furthermore, even among those health-care professionals who may use electronic subscribing, if the system is not followed as designed, such as prescribing via free text, they will not be aware that the medicines they are prescribing are affected by a shortage.

#### Monitor Patients on Alternatives and Reintroduce Initial Therapy

In H-CR, H-SP, and H-SR, monitoring of patients after introducing alternative antibiotic treatment fails owing to non-supportive information technology (IT) systems. For example, in H-SP patient “lab data cannot be cross-checked automatically” and “the system does not allow alerts of clinical issues to pop-up.” “Lack of time and knowledge” on how to appropriately monitor patients is present in H-CR and H-SR, whereas in H-CR, there is a “lack of staff, monitoring Standard Operating Procedures (SOPs), and appropriate IT infrastructure.” In H-BE, sometimes, monitoring or reintroducing the initial treatment is not always an option because of “lack of time and data on shortages” changing on a day-to-day basis. Furthermore, the “fear of over-alerting health-care professionals” and making them reluctant to read emails and check medicine availability also cause additional monitoring to be avoided.

### Hazard Analyses

In H-BE, the highest number of FM (*n* = 5) per sub-process was registered. H-BE also reported the highest number of FM in general (*n* = 16), and H-SP the lowest (*n* = 10). The highest numbers of FM with HSs above 8 were seen in H-CR (*n* = 12) and H-GR (*n* = 11), and the lowest numbers were observed in H-AT and H-BE (Table 2). This led to the identification of 38 FMC with an HS above 8 in H-CR. In H-CR, FM with the highest HS (score of 16) were “information on substitute is not transferred” and “additional patient monitoring not conducted.” In H-AT, five FMs were removed from the analysis that scored above 8 following the decision tree assessment, as the HFMEA teams concluded that there were effective control measures for these hazards. Fifteen FMCs were removed in H-CR following the same methodology. FM and FMC that scored below 8 were mostly included in the analysis in H-AT and H-BE. The highest number of FMCs was observed in H-CR (*n* = 46), and the lowest in H-SP (*n* = 16). H-CR had the highest cumulative FMC HS (cumulative score of 404) and H-AT the lowest (cumulative score of 131). For a list of highly scored FMs across the HFMEA study hospitals, see Table S1. HFMEA research teams mainly opted for control-type corrective measures (e.g., *n* = 22 for H-CR) and eliminate-type measure (e.g., *n* = 23 for H-GE and H-SR).

**Table 2:** Characteristics of hazard analyses between study hospitals.

**Characteristics**	**Hospitals**						**Median [IQR]**
	**H-AT**	**H-BE**	**H-CR**	**H-GR**	**H-SP**	**H-SR**	
Number of sub-processes	6	6	6	6	5	6	6 [5.75–6]
Number of failure modes	11	16	12	13	10	12	12 [10.75–13.75]
Number of failure modes > 8	4	7	12	11	9	10	9.5 [6.25–11.25]
Number of failure modes removed from HFMEA (incl. > 8)	5	4	/	1	2	/	3 [1.25–4.75]
Number of failure modes <8 but included in HFMEA	2	5	/	1	/	2	2 [1.25–4.25]
Number of failure mode causes	21	18	46	31	16	31	26 [17.50–34.75]
Number of failure mode causes > 8	16	10	38	31	15	26	21 [13.75–32.75]
Number of failure mode causes removed from HFMEA (incl. >8)	6	/	15	2	3	2	3 [2–10.5]
Number of failure mode causes <8 but included in HFMEA	3	8	3	/	/	4	3.5 [3–7]
The highest number of failure modes in a sub-process	3	5	3	3	3	2	3 [2.75–3.5]
The lowest number of failure modes in a sub-process	2	1	1	2	1	2	1.5 [1–2]
The highest score for a failure mode	12	12	16	12	9	9	12 [9–13]
Number of actions CONTROL	15	14	22	6	1	6	10 [4.75–16.75]
Number of actions ELIMINATE	/	3	11	23	11	23	11 [7–23]
Number of actions ACCEPT	/	2	/	/	1	/	1.5 [0.75–4.25]

*IQR, interquartile range; SP, sub-process; FM, failure mode; FMC, failure mode causes*.

### Corrective Actions and Hazard Score Reduction

Although assigned severity scores of FM did not statistically differ before and after proposed corrective actions in H-AT, H-BE, and H-SP, they differed significantly in H-CR, H-GR, and H-SR (*p* < 0.005). Median severity, probability, and HSs before and after corrective actions with a total range within each hospital are presented in Table 3. The probability that FM occurred before and after corrective actions did differ significantly in all study hospitals (*p* < 0.005). HSs, based on FM severity and probability, also significantly differed before and after corrective measures (*p* < 0.001). Not only did severity and probability of FM differ significantly within hospitals before and after corrective measures, but they also differed between hospitals (*p* < 0.005; Table 4).

**Table 3:** Failure mode quantification: severity, probability, and hazard scores before and after corrective actions.

	**Median severity score [IQR], (total range)**		***p*-value^**a**^**	**Median probability score [IQR], (total range)**		***p*-value^**a**^**	**Median hazard score [IQR], (total range)**		***p*-value^**a**^**
**Hospitals**	**Before CA**	**After CA**		**Before CA**	**After CA**		**Before CA**	**After CA**	
**H-AT**	3 [2.25–3], (2–3)	3 [2.25–3], (2–3)	n.a.	3 [2.25–4], (1–4)	2 [1–2.75], (1–4)	0.002	8.5 [6.5–9], (3–12)	6 [3–6], (2–9)	0.002
**H-BE**	3 [2.75–3], (2–4)	3 [2–3], (1–4)	0.276	3 [1.75–4], (1–4)	1.5 [1–2.25], (1–3)	0.001	8 [5.5–9], (3–12)	4 [3–6], (2–9)	0.001
**H-CR**	4 [4–4], (4–4)	4 [3–4], (3–4)	0.002	3 [2–4], (1–4)	2 [1–2], (1–3)	<0.001	12 [8–16], (4–16)	6 [4–8], (3–12)	<0.001
**H-GR**	3 [3–4], (2–4)	3 [2.5–4], (2–4)	<0.001	4 [3–4], (3–4)	2 [2–2], (1–2)	<0.001	12 [12–12], (8–16)	6 [4–8], (2–8)	<0.001
**H-SP**	3 [2–3.25], (2–4)	3 [2–3], (2–4)	0.317	4 [2.75–4], (2–4)	2 [1.75–2], (1–2)	0.001	8 [8–9.75], (8–12)	4 [4–6], (3–6)	0.001
**H-SR**	3 [3–3], (3–4)	3 [3–3], (2–3)	0.005	3 [3–3], (2–4)	1 [1–1], (1–2)	<0.001	9 [9–9], (6–12)	3 [3–3], (2–6)	<0.001
**Total**	3 [3–4], (2–4)	3 [3–4], (1–4)	<0.001	3 [2–4], (1–4)	2 [1–2], (1–4)	<0.001	9 [8–12], (3–16)	4 [3–6], (2–12)	<0.001

IQR, interquartile range; CA, corrective actions.

a *p-value estimated by Wilcoxon signed-rank test*.

**Table 4:** Comparison of the severity, probability, and hazard scores among countries.

**Scores**			**Hospitals**						***p*-value^**a**^**
			**H-AT**	**H-BE**	**H-CR**	**H-GR**	**H-SP**	**H-SR**	
**Severity**	Before CA	Mean rank	39.25	47.39	112.50	77.09	45.43	60.36	<0.001
		Median [IQR]	3 [2.25–3]	3 [2.75–3]	4 [4–4]	3 [3–4]	3 [2–3.25]	3 [3–3]	
	After CA	Mean rank	51.63	55.72	105.89	72.05	51.07	58.88	<0.001
		Median [IQR]	3 [2.25–3]	3 [2–3]	4 [3–4]	3 [2.5–4]	3 [2–3]	3 [3–3]	
**Probability**	Before CA	Mean rank	73.13	56.83	61.99	94.24	89.96	59.86	0.001
		Median [IQR]	3 [2.25–4]	3 [1.75–4]	3 [2–4]	4 [3–4]	4 [2.75–4]	3 [3–3]	
	After CA	Mean rank	75.69	70.08	79.50	85.93	78.89	42.14	<0.001
		Median [IQR]	2 [1–2.75]	1.5 [1–2.25]	2 [1–2]	2 [2–2]	2 [1.75–2]	1 [1–1]	
**Hazard**	Before CA	Mean rank	51.16	39.17	83.22	101.36	55.89	65.91	<0.001
		Median [IQR]	8.5 [6.5–9]	8 [5.5–9]	12 [8–16]	12 [12–12]	8 [8–9.75]	9 [9–9]	
	After CA	Mean rank	66.75	55.56	96.14	88.48	68.64	37.83	<0.001
		Median [IQR]	6 [3–6]	4 [3–6]	6 [4–8]	6 [4–8]	4 [4–6]	3 [3–3]	

CA, corrective action.

a *p-value estimated using Kruskal–Wallis test to investigate the difference in dependent variables among countries*.

In all the study hospitals, the proposed hypothetical corrective actions recommended by the HFMEA teams led to HS reductions between 62.5 and 83% (see Table S2). Whereas, H-AT, H-BE, and H-CR mostly opted for “control” measures, H-GR, H-SP, and H-SR opted mainly for “eliminate” measures. Providing more structured communication among health-care professionals and introducing electronic prescribing would both lead to a 66.6% reduction in HSs in H-AT of FM related to the approval and the provision of substitutes. Proactive multi-stakeholder communication and follow-up on shortages could potentially reduce HSs by 83.3% in H-BE when looking at FM related to providing proper alternatives and timely information.

In H-CR, pharmacists more actively participating in the ward would lead to a 66.6% reduction of hazards. Participation would also assist empirical antibiotic treatment by providing patient and antibiotic assessments. Efficient ward stock replenishment would also reduce HSs by 66.6% in H-BE and H-GR for available alternatives to reach the patient. In H-SP, providing an IT system tailored automatically to cross-check patient laboratory data would result in a 62.5% reduction in HSs of the FM relating to failure of monitoring patients on a substitute. In H-SR, standard operating procedures for checking emerging drug–drug interactions after having introduced a substitute, although not in general practice, would reduce HSs by 77.7%.

In H-AT, some corrective actions bore little to no effect on HSs (see Table S3) such as aiming at redistributing tasks of health-care professionals in order to have timely and proper entry of information on available antibiotics into the medicine's ordering system. Moreover, additional patient monitoring after antibiotic substitution in H-BE may not be facilitated by corrective actions such as outsourcing monitoring services or reminding health-care professionals to monitor patients as they show no HS reduction.

## Discussion

### Information Sharing on Antibiotics Affected by Shortages and Their Substitutes

The HFMEA identified 74 FMs in six study hospitals. Fifty-three of these scored 8 or above, representing failures of, in DeRosier's words, “sufficient likelihood to warrant that it be controlled” (41). HFMEA teams took into consideration these failures, which occurred due to lack of timely information on antimicrobial shortages (HS 12 and H-BE) or improper assessment of the information received (HS 9 and H-AT). Because the substitute might not be available to be prescribed/ordered when using the hospital's IT system, such information should be synchronized with availability and be easily accessible via databases instead of time-consuming processes via two-way telephone conversations or email contact. Not knowing the available substitute may jeopardize and delay patient treatment and consequently undermine health outcomes (15, 42, 43).

Five out of the six hospitals report that being able to acquire valid information on shortage duration and availability of alternatives in the initial step. According to Boechenek et al. (44) accessing such information is ultimately determined by the country in which the shortage occurs. In Austria, Belgium, Spain, and Greece, their respective national agencies for medicines are responsible for providing publicly available databases on shortages, whereas in Croatia and Serbia, this falls under the responsibility of the National Health Insurance Fund. However, considering the FMs emerging from this study, it seems that the data provided via these databases in H-AT, H-BE, and H-SP are neither up-to-date nor easily accessible to health-care professionals to readily share information. Reporting itself is not mandatory; thereby, if there is information at all, it is often not comprehensive and does not meet the purpose of finding an alternative. Furthermore, failing to share/transfer the required information on alternatives through internal hospital information systems is evident (FM 9 in H-AT, H-GR, and H-SP but 16 in H-CR), which needs to be better addressed in the future. As indicated, information sharing is essential in potential and ongoing shortages as it can provide appropriate antibiotic substitutions to meet time-sensitive situations. Moreover, established guidelines help restrict antimicrobial use in shortages to those patients who are ranked highest by priority (10). Clear decision-making processes that include communication between hospital pharmacies and DTCs may therefore assist through prioritizing patients and implementing substitution protocols (45). Health-care professionals should also be educated on how to use these protocols and how to prioritize patients when needed (3).

A lack of timely and direct communication represents one of the main barriers in managing antibiotic shortages (4, 46). Arising from the features of IT facilities coupled with a lack of human resources in conjunction with the high workload described, the FMs are linked to the absence of proper channels of communication scored above 9 in H-BE, H-GR, and H-SP. Moreover, in H-BE, where the FM scored 9, a delay in transferring data on antibiotic shortages led to unadjusted stock levels. To combat against these issues, it is essential to disseminate information on shortages successfully. Therefore, a network of decision-making clinicians and other stakeholders within a respective health-care setting must be established (10). Notifying health-care professionals should be performed prior to the actual medicine request submitted to the pharmacy when the antibiotics in question are only available in limited quantities or otherwise completely unavailable (10). Furthermore, any notification on shortages should also comprise additional data on substitutes, including their indication for use, and should be readily available at the point of care. These notifications must be compiled as such so that health-care professionals need not perform any additional literature research when trying to decide on alternatives (10, 17). The decision-making process may also be based on checklists to facilitate consistency in substitution, where data regarding a medicine's dosage, administration particularities, and any potential look-alike or sound-alike risks are to be clearly stated (16).

### Appropriateness Assessment for Antibiotic Substitution

Multidisciplinary committees play a vital role in facilitating decisions on antibiotics within a health-care facility (4). DTCs in both H-AT and H-BE oversee the process of substitution, thereby allowing for a multidisciplinary patient assessment with particulars to given cases to be accounted for. As presented in H-BE, an antimicrobial stewardship subcommittee thoroughly elaborates on aspects of antimicrobial substitution and accounts for potential patient groups and diseases. Antimicrobial stewardship programs have also been cited as a way to disseminate information on substitution in order to provide better-coordinated care and increase patient safety (10, 47).

Information assessment on a substitute in health-care settings did not appear to follow a standardized manner in our study, which may lead to potential misinterpretation of the information on administration patterns of alternatives. To illustrate this, in H-SR and H-CR, a suitable antimicrobial alternative is primarily based on a structured assessment of the microbiology strain's characteristics and the alternative spectrum of activity available. However, insufficient access to information on the availability of suitable substitutes aggravates existing FMs of not having a review of alternative antibacterial spectra in H-SR (FM 9) or adverse events profile in H-CR (FM 12).

“Inter-professional and interpersonal miscommunication” on roles may create health-care professionals' perception that mitigation of shortages is not in their scope of activities causing 3/11 FMs in H-AT and 3/13 FMs in H-GR. When deciding on substitutes in H-GR, difficulties in accessing patient data and by an “absence of health-care professionals' willingness to provide additional information” both form obstacles to some of the hospital pharmacists. If an alternative's reconstitution and administration pattern are not fully entered into the system, it represents another challenge to less experienced unsupervised personnel (H-SP).

Underlined by Griffith et al. (10), pharmacists dedicated to antibiotic stewardship must carry out a medicine review in order to find the most suitable antimicrobials in case no suggestions via guidelines are available (10). Moreover, this may be conducted by a team able to assess available antibiograms for a respective health-care setting or determine empiric antibiotics (48).

### Access to and Validation of Patient Medical Records

Patient-record validation represents one of the substitution sub-processes in all six study hospitals. Nonetheless, the HFMEA depicted numerous FMCs within this sub-process, demonstrating an absence of clear guidelines on validation as part of the substitution. FMs emerging from not fully validated patient medical records prior to a substitution demonstrate the need to account for the clinical status of a patient (FM > 8 in H-GR, H-CR, and H-SR). The majority of failures come directly or indirectly from the “absence of electronic prescribing,” which itself is a major barrier in the efficient assessment of patient data and safe substitution practices. Computerized physician order entry (CPOE) does reduce errors and makes prescribing a safer process, as confirmed by the HFMEA (49). Decisions on alternatives in H-SP distinctly noted the need for providing more thorough laboratory hepatic and renal parameters automatically screened for in medical records during the decision process on alternatives, as well as for continuous monitoring throughout the patient's treatment.

### Staff Scarcity and Time Constraints in Antibiotic Substitution

“Lack of time” proved to be an FMC in more than half of the sub-processes of antibiotic substitution in H-AT, H-CR, H-GR, H-SP, and H-SR, leading to 5/11 FMs (45%), 2/12 FMs (17%), 5/13 FMs (38%), 4/10 FMs (40%), and 6/12 FMs (50%), respectively. Managing shortages is often seen as solely the task of a hospital pharmacist, confirmed by studies conducted in Belgium and the EAHP where hospital pharmacists are reported to spend a significant time mitigating shortages (2, 44).

Time is a key factor in prescribing medicine. Griffith et al. note that any delays in treatment and care can cascade into higher safety risks at each subsequent stage of antimicrobial treatment (42). Therefore, when the prescriber is unfamiliar with a substitute due to time constraints, it bears a negative influence on the subsequent steps within treatment, which may result in being less aware or a proper alternative's dosage requirements and contraindications for use (45). Clinicians therefore need to be highly trained to provide effective treatment when dealing with priority group patients to provide the right medicine promptly to avoid unnecessary delays (10). To illustrate, for those patients with nosocomial infections (e.g., pneumonia, blood stream infection, and sepsis), a timely provided substitute is essential as it lowers risks of mortality (42, 50–58). Moreover, optimal antimicrobial use is critical owing to an increasing number of multiresistant strains, which only respond to a narrow range of antibiotics (42).

Apart from time as a distinct issue, “scarcity of hospital pharmacy staff” is pronounced in H-SR and H-CR, accounting for 4/12 (33%) of the FMs as they have a lower staff number. These failures are in line with the WHO's report predicting the ongoing global shortage of health-care professionals to reach 18 million professionals worldwide by 2030 (59). As such, scarcity is also an issue in hospitals employing a larger staff as well (H-AT, H-GR, and H-SP). Partially owing to a “lack of time” and “task prioritization,” health-care professionals do not view “the assessment of antibiotic alternative's appropriateness” as a priority and part of “everyday practice” (H-GR, H-SP, H-CR, and H-SR). Consequently, proper patient clinical status assessments and patient safety could be jeopardized when trying to find a suitable alternative. Inappropriate medicine management also further adds to risk by limiting the ability of health-care professionals to assess the individual patient's needs in a shortage (2).

When staff or time is short, it limits the ability of health-care professionals to find an appropriate alternative. Therefore, alternatives should be established in advance for critical medicines used in the ER and other departments because they are most often affected by shortages (2, 7, 45, 60). When substituting a medicine, it is necessary to have experienced hospital pharmacists perform accurate calculations (4).

### Monitoring and Communication With a Patient Throughout Antibiotic Shortages

In the literature, the focus has shifted from barely reporting and listing shortages to characterizing their impact on patient safety and the harm caused. The literature also proposes a variety of management “strategies,” which are highly needed, as confirmed by findings from the present research (47, 61). Gundlapalli et al. (15) highlight the need for new modalities of managing shortages to be necessary because practitioners are now able to gather data on shortages more quickly than previously (15). However, disseminating data on the clinical impact where insufficient antibiotics substantially affect treatment is still missing (15). Substitution therefore depends on the aggregated data based on the extent of shortages as well as their impact on clinical practice and potential solutions of dealing with the shortage (10, 15).

The HFMEA bears out the need for more effective strategies to overcome shortages through data sharing and monitoring. When an alternative antibiotic is introduced, several research HFMEA study teams had highly scored FMs related to the absence of patient monitoring (H-SP-9, H-SR-9, and H-CR-16, but 12 under partially conducted monitoring). Insufficient checking of the patient's medical records in H-GR (HS = 12) and H-SP (HS = 9) were also found.

When introduced, the patient must be additionally monitored as the substitute might not be the best possible therapeutic solution (62). Patient monitoring of alternatives was shown in the HFMEA to be regularly conducted in H-BE (allowing for time). Whereas, in H-CR, H-SP, and H-SR, interlinked FMCs including a lack of staff, IT support, and available laboratory software for data to be cross-checked (H-SP) hindered this type of monitoring. Antimicrobial stewardship programs, facilitated by electronically led medical records, are also needed to support continuous, effective monitoring and evaluation of patient health outcomes after substitution takes place in order to provide corrective measures in time if needed (63). Corrective actions are avoidable if patients stay under supervision during substitution, allowing follow-up on the effectiveness of the new therapeutic regime.

The FMC “insufficient competency in communication among health-care professionals” scored 8 in H-CR, presenting a barrier in communicating/discussing with patients on antimicrobial shortages. Patients themselves were also found to be limited in their willingness to be informed on therapies. Moreover, physicians/pharmacists did not communicate effectively to the patient when deciding on a substitute. This comes in stark contrast to the recommendation that patients have to be informed on potential risks emerging from potentially less efficient or more toxic provided alternatives, at least when the clinical situation allows communication with the patient (64).

### Health-Care Failure Mode and Effect Analysis-Team Corrective Measures for Reducing Hazard Scores

The HFMEA teams generally opted for initiating corrective actions in their hospitals. These imply the implementation of training courses and the sensitization of health-care professionals to the management of antibiotic shortages, introducing internal SOPs and improving communication. Such changes would hypothetically reduce HSs by more than 60%. Except for H-SP, all study hospitals had no specific shortage-management task force group in place. Moreover, there were no existing internal guidelines providing strategies on how to manage shortages in all hospitals; this was under development in H-BE.

The H-AT team claimed that more structured communication among health-care professionals would reduce scores by 66%. It was also reported that a more proactive communication on a multi-stakeholder platform would lower HSs by 83%, whereas the more prominent role of ward pharmacists on shortages in H-CR would also cut scores by 66%. In H-SP, corrective actions also included the proposal of an advanced IT supported program for cross-checking laboratory data for all patients monitored after substitution. Doing so would likely reduce HSs by 63%. According to Erin Fox of the US Drug Information Center from the University of Utah, it is necessary to have a process in place defining an alternative's approval and ethical considerations for a substitute's allocation that has already been established prior to a shortage occurring. Moreover, this entails information on how to gather sufficient data on a shortage, to purchase alternatives, to assess storage conditions, to apply procedures for preparing and dispensing the medicine, to decide whether to reallocate substitutes, to implement IT changes, and to actively communicate all of these parameters (4).

### Study Limitations and Potential

Applying the HFMEA in a health-care setting bears a certain subjectivity, as seen in the selection of particular FMs and the manner in which the HFMEA teams use their subjective perception to appoint scores. Nonetheless, having a multidisciplinary team alleviates the bias in risk assessment aiming at assuring an objective perception of the processes, sub-processes, FMs, and failure causes through a consensus of team members. Additionally, all assessments and meetings were supervised by one member of the research team (NM; lead) to reduce variability in interpretation. Although the HFMEA results from one hospital to another may not easily be extrapolated to other settings, given the particularities of each health-care setting, this study highlighted that the HFMEA in medicine shortages provided a number of similarities in detected FMs and causes. Moreover, the process used in each hospital was generalizable in terms of defined overarching FMs and patterns emerging from this risk assessment, which can assist in preventing the aforementioned risks from occurring in other health-care settings. It must be distinctly noted that all hospitals where the HFMEA took place are university-teaching hospitals, which might bear on how antimicrobial shortages are managed, as compared with non-teaching hospitals. It is generally cited that teaching hospitals are more efficient and have better quality of care than non-teaching (65). As such, their teaching status may account for having health-care professionals possessing greater experience in treating certain conditions, which may account for better patient outcomes (66). However, other factors such organizational culture, adopting health technologies in hospitals, and the health-care staff available may all result in better health-care provided (67). For this reason, it is difficult to distinguish better patient satisfaction and health outcomes in terms of treatment appropriateness and effectiveness when it is a teaching institution (68, 69). Another limitation of the study was not having a patient representative involved in the assessments, meaning that patients' perceptions on antibiotic substitution stemming from shortages could not be considered. In addition, considering that only hypothetical corrective actions emerging from this HFMEA have here been analyzed and displayed in each study hospital, the real HS reduction and actual impact on everyday practice needs to be evaluated in subsequent prospective risk assessment analyses in each respective hospital in order to fully assess their impact on risk reduction. In the meantime, we believe that this analysis on the basis of a robust methodology will be helpful to all European hospitals to aid them in reducing the impact of antimicrobial shortages in the future.

## Conclusion

Through a prospective risk assessment of antibiotic substitution following shortages, a variety of corrective actions were identified theoretically proven to decrease the risks. To the best of the authors' knowledge, this is the first HFMEA study conducted specifically on antimicrobial substitution processes among European hospitals. Our study shows an absence of a structured approach in addressing all risks emerging from antibiotic substitutions, including access and provision of information needed to deliver an alternative treatment in a timely manner, among European hospitals. The data obtained via HFMEA confirm that communication plays a crucial role in successfully mitigating risk during substitution following shortages. Suggestions made by the HFMEA teams are mainly oriented toward improving internal and external communication via IT systems and through the thorough revision of existing mitigating procedures. Additional suggestions include recommending the introduction of new protocols for antibiotic substitution when shortages arise, as there are appears to be no existing internal guidelines in place for each study hospital. A similar situation is likely to exist in an appreciable number of other European hospitals. These guidelines should clearly describe steps on how to promptly react to antibiotic or any other medicine shortages, emphasizing the preparedness of health-care professionals and possible preventable actions that could be carried out to help avoid patient harm. The scarcity of data on the availability of antibiotics hinders health-care professional's efforts to conduct timely assessment of potential alternatives to address current patient needs. Nevertheless, as shown throughout the analysis, this study does indicate that health-care professionals are managing to use existing IT and human resources, despite all obstacles, to secure optimal antimicrobial treatment for patients and control risks. This can be built on in the future to further improve patient care.

## Data Availability Statement

The datasets generated for this study are available on request to the corresponding author.

## Author Contributions

NM designed and carried out the study using existing HFMEA methodology, compiled the first draft and the subsequent iterations of the manuscript. NM and the following authors coordinated HFMEA teams and contributed to data collection carried out in the hospitals: KT, AA, AN, CY, LP, GS, DK-P, and TD. NM and MK performed data analysis and interpretation needed for the manuscript. BG, BM, EO, IH, and TB contributed to critical analysis and interpretation of data as well as revised the manuscript. All authors read, commented on, contributed to the manuscript for the accuracy of the content, finally approved the version to be published and agreed to be accountable for all aspects of the work in terms of its accuracy and integrity.

## Conflict of Interest

The authors declare that the research was conducted in the absence of any commercial or financial relationships that could be construed as a potential conflict of interest.
