# Supplementary material for: Risks in Antibiotic Substitution Following Medicine Shortage: A Health-Care Failure Mode and Effect Analysis of Six European Hospitals
Source: Front Med (Lausanne). 2020 May 12;7:157. doi: 10.3389/fmed.2020.00157 (PMC7235345; doi:10.3389/fmed.2020.00157)
Supplement: Supplementary file 1 [file Table_1.DOC]

**Тable S1**. Highly scored failure modes across HFMEA study hospitals

| **Hospital** | **Highly-scored failure modes (extracted from participant responses)** | **Probability** | **Severity** | **Hazard Score** |
| --- | --- | --- | --- | --- |
| **H-AT** | *“not properly checking the information on shortages”* | **3** | **3** | **9** |
|  | *“not getting approval for the appropriate substitute”* | **3** | **3** | **9** |
|  | *“not placing the information in the ordering system”* | **3** | **3** | **9** |
|  | *“additional information on a substitute is not digested properly”* | **3** | **3** | **9** |
| **H-BE** | *“not getting timely information on shortages”* | **3** | **4** | **12** |
|  | *“wrong format of communication”* | **3** | **3** | **9** |
|  | *“not timely adjustment of hospital stock”* | **3** | **3** | **9** |
| **H-CR** | *“substitute adverse event profile not evaluated at all”* | **3** | **4** | **12** |
|  | *“substitute adverse event profile not evaluated in real time”* | **4** | **3** | **12** |
|  | *“information on substitute is not transferred”* | **4** | **4** | **16** |
|  | *“additional patient monitoring not conducted”* | **4** | **4** | **16** |
|  | *“additional patient monitoring conducted partially”* | **4** | **3** | **12** |
| **H-GR** | *“not checking the patient medical record”* | **4** | **3** | **12** |
|  | *“checking the patient medical record incorrectly”* | **3** | **4** | **12** |
|  | *“not checking alternative routes of administration of the substitute”* | **4** | **3** | **12** |
|  | *“not passing the information on alternative’s route of administration to other healthcare professionals”* | **3** | **4** | **12** |
|  | *“not having the proper communication channels”* | **3** | **4** | **12** |
|  | *“communication with the persons not in charge of the therapy”* | **3** | **4** | **12** |
| **H-SP** | *“available substitutes not provided via IT system in due time”* | **3** | **3** | **9** |
|  | *“patient records not validated before substitution takes place”* | **3** | **3** | **9** |
|  | *“healthcare professionals only partially informed on substitution”* | **3** | **3** | **9** |
|  | *“monitoring not properly taking place after substitution”* | **3** | **3** | **9** |
| **H-SR** | *“not reviewing alternative’s spectrum of activity”* | **3** | **3** | **9** |
|  | *“not reviewing dosage, routes of administration and stability“* | **3** | **3** | **9** |
|  | *“not reviewing the need for aditional monitoring“* | **3** | **3** | **9** |
