# Supplementary material for: Risks in Antibiotic Substitution Following Medicine Shortage: A Health-Care Failure Mode and Effect Analysis of Six European Hospitals
Source: Front Med (Lausanne). 2020 May 12;7:157. doi: 10.3389/fmed.2020.00157 (PMC7235345; doi:10.3389/fmed.2020.00157)
Supplement: Supplementary file 2 [file Table_2.DOC]

| **Hospital**  **Table S2. Major hazard score reduction based on corrective actions in study hospitals** | **Failure mode** | **Failure mode cause** | **Type of HCA** | **HCA** | **Process/Outcome measure** | **Person responsible** | **HS before** | **HS after** | **HS reduction (%)** |
| --- | --- | --- | --- | --- | --- | --- | --- | --- | --- |
| **H-AT** | **Not obtianing approval for an appropriate substitute** | **Lack of time** | **Control** | **Re-evaluate task distribution** | **Number of effectively conducted tasks** | **Head of Pharmacy** | **9** | **3** | **66.6** |
|  |  | **Wrong means of communication** | **Control** | **Provide more structured procedures for communication** | **Number of effective communication sessions** | **Head of Pharmacy** | **9** | **3** | **66.6** |
|  | **Available substitute is not prescribed to a patient** | **Physician not well informed/misinformed about the substitute; not considers a priority finding the wright information** | **Control** | **Electronic prescribing** | **Number of misinformed** | **IT/Management** | **9** | **3** | **66.6** |
| **H-BE** | **Not getting the timely information on shortages** | **The manufacturer/wholesaler is not willing to disclose the right information on time** | **Control** | **Proactive follow-up of potential shortages** | **Number of timely reported shortages** | **Procurement pharmacist** | **12** | **2** | **83.3** |
|  | **A specific treatment regimen for one or more groups of patients is not taken into account when choosing an alternative** | **Inaccurate information from the manufacturer related to indication; market situation** | **Control** | **Implementing proactive communication strategy with the industry/wholesalers** | **Number of accurate information from the industry/wholesalers on a monthly level** | **Head of pharmacy/Procurement pharmacist** | **12** | **2** | **83.3** |
|  | **Replenishment of the ward stock not performed in a timely manner** | **Absence of obligatory ward stock replenishments on a daily bases** | **Control** | **Force replenishment on a specific day as soon as product arrive** | **Number of days a ward is out of a suggested alternative** | **Head of Pharmacy/Procurement pharmacist** | **9** | **3** | **66.6** |
| **H-CR** | **Review of antibiotic spectrum not conducted** | **Empirical treatment considered only** | **Control** | **Pharmacist actively participate at the ward rounds; education of HCPs** | **Appropriateness of antibiotic usage; the length of hospitalization; education evaluation** | **Head of pharmacy; Hospital management; Head of wards** | **12** | **4** | **66.6** |
|  | **Administration not properly conducted in terms of reconstitution (compatibility with solvents) and route of administration** | **Staff not properly informed-Inter-professional transfer of information not properly conducted** | **Control** | **Written transfer of information; IT supported notification pop-up on change of antibiotic** | **Less uninformed staff; reduction of errors occurring in information transfer** | **Head of pharmacy; Hospital management; Head of wards** | **12** | **4** | **66.6** |
| **H-GR** | **Partially checking the availability of the alternative medicines** | **Not checking ward stocks** | **Eliminate** | **SOP for daily ward stock assessment** | **Efficient usage of available ward stocks** | **Hospital Head Nurse, Pharmacy Department** | **9** | **3** | **66.6** |
|  | **No checking of the pharmacoeconomic aspects of the substitute/alternative medicine** | **Lack of pharmacoeconomic data on the substitute** | **Eliminate** | **Provide full access to pharmacoeconomic data on substitutes** | **Sufficient pharmacoeconomic data on proposed substitute** | **IT Department/Pharmacy Department** | **12** | **4** | **66.6** |
|  |  | **Personal perception of how suitable the substitute is** | **Eliminate** | **Clear pharmacoeconomic analysis guideline** | **Objective substitution assessment** | **Pharmacy Department/Ward/Physicians** | **12** | **2** | **83.3** |
|  | **Not having the proper communication channels** | **Inter-professional misunderstanding** | **Eliminate** | **Intensified multidisciplinary collaboration** | **Well established inter-professional communication** | **Quality/Head of Wards/Head of Pharmacy** | **12** | **4** | **66.6** |
| **H-SP** | **Monitoring of a patient after introducing a substitute medicine is not properly carried out by a pharmacist** | **IT system does not allow active monitoring concerning lab data (renal; liver function), a pharmacist is not able to monitor patients routinely regardless of their initial renal/liver status (not only those patients with known renal impairment)** | **Eliminate** | **Provide IT support as a tool, which actively cross-check lab data (renal/liver function) and prescribed medicines and easily displays results to a pharmacist** | **Conducted IT cross-check verifications of lab renal/liver data and prescribed medicines** | **Head of IT department/Head of Pharmacy Department/Head of Quality Department** | **8** | **3** | **62.5** |
| **H-SR** | **AB spectrum not reviewed** | **Lack of time** | **Control** | **Efficient time management** | **Sufficient/average time needed to check the AB spectrum of activity** | **Head of Hospital Pharmacy/Ward** | **9** | **3** | **66.6** |
|  | **AB spectrum not properly reviewed** | **No available proper literature** | **Eliminate** | **Provide access to up-to-date literature** | **Number of successful data searches** | **Head of Hospital Pharmacy/Ward** | **9** | **3** | **66.6** |
|  | **Information on dosage regimen, stability and route of administration not properly reviewed** | **Not considered as a priority** | **Control** | **Create SOP** | **Number of successfully followed procedures** | **Head of Hospital Pharmacy/Ward** | **9** | **3** | **66.6** |
|  | **Data on dosage regimen misinterpreted** | **Low quality of information** | **Eliminate** | **Provide precise available data** | **Number of used adequate data sources** | **Hospital Pharmacy/Ward** | **6** | **2** | **66.6** |
|  |  | **Low inter-professional communication** | **Control** | **Acquiring techniques for more efficient communication** | **Processes involving official communication regarding dosage regimen requirements** | **Hospital Pharmacy/Ward/Microbiology** | **9** | **3** | **66.6** |
|  | **Effectiveness of original versus alternative AB not reviewed** | **Lack of time** | **Control** | **Efficient time management** | **Sufficient/average time needed to check the AB spectrum of activity** | **Head of Hospital Pharmacy/Ward** | **9** | **3** | **66.6** |
|  |  | **Not considered as a priority** | **Control** | **Create SOP** | **Number of successfully followed procedures** | **Head of Hospital Pharmacy/Ward** | **9** | **3** | **66.6** |
|  |  | **Not part of everyday practice** | **Control** | **Create SOP** | **Number of successfully followed procedures** | **Head of Hospital Pharmacy/Ward** | **9** | **3** | **66.6** |
|  | **Review for additional patient monitoring when substituting an original medicine with an alternative medicine not conducted** | **Lack of time** | **Control** | **Efficient time management** | **Sufficient/average time needed to check the need for monitoring** | **Head of Hospital Pharmacy/Ward** | **9** | **3** | **66.6** |
|  |  | **Not considered to be the part of a routine practice** | **Control** | **Create SOP** | **Number of successfully followed procedures** | **Head of Hospital Pharmacy/Ward** | **9** | **3** | **66.6** |
|  | **The potential for newly occurred interaction not reviewed before an alternative treatment is introduced** | **Not part of the routine practice** | **Control** | **Create SOP** | **Number of successfully followed procedures** | **Head of Hospital Pharmacy/Ward** | **9** | **2** | **77.7** |
|  |  | **Lack of time** | **Control** | **Efficient time management** | **Sufficient/average time needed to check the need for monitoring** | **Head of Hospital Pharmacy/Ward** | **9** | **3** | **66.6** |
|  |  | **Not considered to be a priority** | **Control** | **Create SOP** | **Number of successfully followed procedures** | **Head of Hospital Pharmacy/Ward** | **9** | **2** | **77.7** |
|  | **Review of potential interactions when an alternative medicine is introduced not properly conducted** | **No access to information on medicines interactions** | **Control** | **Provide access to electronic data bases** | **Number of visits to a data base** | **Head oh Pharmacy/Head of Ward/IT services** | **9** | **3** | **66.6** |
|  |  | **Access only to obsolete paper-based information** | **Control** | **Provide access to electronic data bases** | **Number of visits to a data base** | **Head oh Pharmacy/Head of Ward/IT services** | **9** | **3** | **66.6** |
|  |  | **No online interaction checker provided to healthcare professionals** | **Control** | **Intergrade online interactions checker with the hospital IT system** | **Number of detected clinically significant interactions** | **Head oh Pharmacy/Head of Ward/IT services** | **9** | **3** | **66.6** |

AB, antibiotic; HCA, hypothetical corrective action; HCPs, healthcare professionals; HS, hazard score; IT, information technology; SOPs, standard operating procedures.
