# Supplementary material for: Risks in Antibiotic Substitution Following Medicine Shortage: A Health-Care Failure Mode and Effect Analysis of Six European Hospitals
Source: Front Med (Lausanne). 2020 May 12;7:157. doi: 10.3389/fmed.2020.00157 (PMC7235345; doi:10.3389/fmed.2020.00157)
Supplement: Supplementary file 3 [file Table_3.DOC]

**Table S3. Hazard scores with no reduction based on corrective actions in study hospitals**

| **Hospital** | **Failure mode** | **Failure mode cause** | **Type of HCA** | **HCA** | **Process/Outcome Measure** | **Person responsible** | **HS before** | **HS after** |
| --- | --- | --- | --- | --- | --- | --- | --- | --- |
| **H-AT** | **Information placed in the ordering system is delayed** | **High workload** | **Control** | **Redistribute tasks in a specific area of expertise** | **Number of redistributed tasks in order to have less workload** | **Head of Pharmacy** | **8** | **8** |
|  | **Not placing the information in the ordering system** | **High workload** | **Control** | **Redistribute tasks in a specific area of expertise** | **Number of redistributed tasks in order to have less workload** | **Head of Pharmacy** | **9** | **9** |
| **H-BE** | **No timely adjustment of stock at hospital ward** | **Due to not foreseen supply and medicines market problems.** | **Accept** | **Performing ongoing analysis taking into account market realities** | **Number of successfully mitigated shortages based on ABS committee decisions** | **Head of ABS Committee/Head of Pharmacy** | **9** | **9** |
|  | **Information on medication use sent throughout the hospital is not correct/incomplete** | **Wrong information sent based on misinterpreted information; possible mistake in drafting the written information** | **Eliminate** | **Introduce text double check control by the second pharmacists** | **Number of wrong information provided** | **Head of Pharmacy/Head of ABS Committee** | **4** | **4** |
|  | **Information on additional monitoring after substitution is introduced and not properly transferred** | **Doctors are not aware of needed TDM** | **Control** | **Double check by another reference physician to evaluate the need for TDM** | **Number of needed and non-conducted TDM analysis** | **Head of Wards/Head of Pharmacy/Head of ABS Committee** | **3** | **3** |
|  |  | **No access to the test** | **Accept** | **Explore the possibility to perform TDM in another clinics** | **Number of needed and non-conducted TDM analysis** | **Head of Wards/Head of Pharmacy/Head of ABS Committee** | **3** | **3** |
|  |  | **HCPs forgot about the test** | **Control** | **Create a pop-up alert in CPOE, or poster presentation in the ward for medicines where TDM is needed** | **Number of needed and non-conducted TDM analysis** | **Head of Wards/Head of Pharmacy/Head of ABS Committee** | **3** | **3** |

ABS, antibiotic stewardship; CPOE, computerized physician order entry; HCA, hypothetical corrective action; HCPs, healthcare professionals; HS, hazard score; SOPs, standard operating procedures; TDM, therapeutic drug monitoring.
